# Retrospective analysis of the occurrence, potential risk factors and medical significance of pulmonary complications after total shoulder arthroplasty from the National Inpatient Sample database (2010–2019)

**DOI:** 10.1186/s13741-024-00490-9

**Published:** 2025-01-10

**Authors:** Mengning Dong, Huitong Liang, Jinlang Fu, Zeying Guo, Hao Xie, Qinfeng Yang, Qingmei Yu, Xiaomin Hou

**Affiliations:** 1 Department of Anesthesiology, Nanfang Hospital, Southern Medical University, Guangzhou, 510515 Guangdong China; 2 Division of Orthopaedic Surgery, Department of Orthopaedics, Nanfang Hospital, Southern Medical University, Guangzhou, 510515 Guangdong China; 3 The First Clinical Medical School, Guangdong Medical University, Zhanjiang, 524023 Guangdong China; 4 Division of Orthopaedics and Traumatology, Department of Orthopaedics, Nanfang Hospital, Southern Medical University, Guangzhou, 510515 Guangdong China

## Abstract

**Background:**

In USA, total shoulder arthroplasty (TSA) ranks amongst the top five surgeries that require hospitalization. As a result, the healthcare system in USA could face a considerable financial strain due to the emergence of subsequent pulmonary problems. This study aimed to conduct a thorough examination of the prevalence, influential factors and medical importance of pulmonary complications, with emphasis on pneumonia, respiratory failure and pulmonary embolism (PE) following total shoulder arthroplasty (TSA) procedures in USA.

**Methods:**

The National Inpatient Sample (NIS) was utilized to survey all patients who underwent primary elective TSA from 2010 to 2019. Pneumonia, respiratory failure and PE following TSA were considered to be pulmonary consequences. The inpatient expenses, length of hospitalization, death rates and patient characteristics of those with and without reported perioperative pulmonary problems were compared. The utilization of trend weights was necessary to obtain incidence estimates across USA, considering the stratified framework of the NIS database and the dependence on observed frequencies within the database. Two assessments were utilized to assess the projected annual rates of complications.

**Results:**

Between 2010 and 2019, a total of 189,695 patients were estimated to underwent primary elective TSA. Infections, such as pneumonia, respiratory failure or PE, complicated 1.4% (95% CI, 1.52%–1.64%) of TSA operations. The ailments at this period that were most likely to result in pulmonary problems were ulcer (adjusted odds ratio [AOR] = 9.43; 95% CI, 4.99–46.91), pulmonary circulation disorders (AOR = 9.01; 95% CI, 4.56– 31.92), weight loss (AOR = 4.84; 95% CI, 2.15–10.88), fluid and electrolyte disorders (AOR = 3.55; 95% CI, 2.55–4.95), alcohol abuse (AOR = 1.56; 95% CI, 1.08–2.26), congestive heart failure (AOR = 3.09; 95% CI, 1.83–5.24), chronic pulmonary disease (AOR = 2.45; 95% CI, 1.60–3.75), deficiency anaemia (AOR = 1.56; 95% CI, 1.08–2.26), depression (AOR = 1.47; 95% CI, 1.03–2.11) and obesity (AOR = 1.46; 95% CI, 1.01–2.11). A correlation was found between perioperative pulmonary problems and extended LOS (+ 3 days; 95% CI, 2–6) and increased hospitalization costs (= + 20,514 US dollars; 95% CI, 14,109–35,281).

**Conclusions:**

This investigation primarily aimed to ascertain potential risk factors linked to pulmonary issues that may occur after TSA. The analysis revealed that the pneumonia rates decreased each year, whereas the PE rates remained relatively stable. A noticeable and consistent increase was found in respiratory failure from 2010 to 2019. The findings suggests that individuals who are older (primarily between the ages of 60 and 80 years) and female exhibit increased rates. These factors could help stratify patients and reduce the risk of potential complications. This claim is especially applicable in PE because it is associated with more significant improvements in resource utilization.

## Background

In USA, more than 50,000 individuals undergo total shoulder arthroplasty (TSA) annually, with its prevalence increasing faster than that of lower-extremity arthroplasty procedures (Valsamis et al. 2023). The use of TSA continues to increase with population aging, expanded indications and the advancement of technology (Cancienne et al. 2017; Lu et al. 2020; Theodoulou et al. 2019). From 2011 to 2030, the demand for primary TSA is anticipated to increase by 333.3% in patients aged 55 years and by 755.4% in individuals older than 55 years (Kim et al. 2011; Jain et al. 2006). However, problems arise in roughly 10% of individuals, encompassing venous thromboembolism, cardiac issues and potentially fatal medical complications (Bohsali et al. 2017; Ma et al. 2021; Singh et al. 2011), which could impose a significant load on the healthcare system (Malcolm et al. 2020).

Pulmonary complications, such as pneumonia, respiratory failure and pulmonary embolism (PE), represent significant and possibly life-threatening events following total joint arthroplasty (Lee et al. 2019). Postoperative pneumonia can result in substantial morbidity and mortality as a serious complication in the perioperative period (Ephgrave et al. 1993; Schein 2002; Thompson et al. 2006). A notable correlation exists between pneumonia and a substantial increase in perioperative mortality, with a magnitude exceeding 30-fold, as well as a substantial increase in readmission rates following total joint arthroplasty (TJA), reaching a magnitude of 16-fold (Bohl et al. 2017). Postoperative respiratory failure is regarded as a noteworthy patient safety metric owing to its correlation with a considerable 26-fold increase in mortality rates within 30 days following general, orthopaedic, neurosurgical, urological, gynaecological and obstetric surgical procedures (Johnson et al. 2007). According to authoritative studies, the yearly occurrence of PE range from 750 to 2690 instances per 100,000 individuals, and perioperative mortality as high as 28% has been described (Young et al. 2015). Even healthy patients who actively receive anticoagulant therapy receiving remain at risk for PE development (Lieberman et al. 2017).

Tennison et al. investigated the occurrence, determinants and consequential implications of pulmonary problems following total knee arthroplasty (TKA) and total hip arthroplasty (THA) procedures in USA. The investigations were conducted independently for each procedure (Malcolm et al. 2020). Significant variations in adverse postoperative outcomes were found despite the similarities between these operations (Hespanhol and Bárbara 2020). The findings may not be generalisable to TSA population. From an anatomical and surgical perspective, the dissimilarities amongst the hip, knee and shoulder make it impossible to accurately extend the findings of lower extremity arthroplasty to TSA population (Collins et al. 2019). Since the rate of TSA and the prevalence of perioperative pulmonary complications have been steadily increasing, the significance of documenting the occurrence, possible variables contributing to risk and treatment implications of pulmonary problems after TSA in USA cannot be overemphasized (Kim et al. 2011; Hespanhol and Bárbara 2020; Day et al. 2010; Padegimas et al. 2015). This approach has the potential to enhance preoperative optimisation efforts and improve patient stratification, hence reducing the risk of potential problems (Young et al. 2015; Smucny et al. 2015; Lung et al. 2019).

Twenty percent of all discharges from non-federal, short-term, general and other specialty hospitals in USA are included in the stratified National Inpatient Sample (NIS) database (Healthcare Cost and Utilization Project (HCUP) Statistical Briefs 2006). NIS is an ideal choice for estimating disease trends, surgical prevalence and perioperative outcomes in USA because it is the openly available, nationwide and the largest fully paid inpatient care database (Magill et al. 2014). NIS could be used to describe and analyse the prevalence, risk factors and clinical implications of respiratory problems following TSA.

## Methods

### Data source

The NIS database is the largest fully paid inpatient database in USA. As part of the Health Care Cost and Utilization Project (HCUP) and the Institute for Health Care Research (IHR), including general, short-term, non-federal and other specialty hospitals, NIS includes a stratified sample of discharges from over 1000 hospitals throughout USA. The provided sample represents an estimated 20% of the total yearly hospitalizations in the country (Rhemtulla et al. 2021). The data provided includes sampling weights used to calculate prevalence estimates for diseases across the country. Data can be extracted from the database, encompassing patient profiles, service levels, overall hospital expenditures, and diagnostic codes in accordance with the International Classification of Diseases, Ninth Revision, Clinical Modification ([ICD-9-CM] and ICD-10-CM) clinical modification.

### Study Population/Patient selection

The diagnostic and procedural information used in this study was derived from the ICD-9-CM and ICD-10-CM coding systems. This study aimed to conduct a comprehensive data query of the NIS database, focusing on main TSA cases that occurred between 2010 and 2019. Specifically, instances where the primary procedure code recorded were 81.80/81.81/81.88/81.97 (ICD-9-CM) and 0RRJ00Z/0RRJ07Z/0RRJ0J6/0RRJ0J7/0RRJ0JZ/0RRJ0KZ/0RRK00Z/0RRK07Z/0RRK0J6/0RRK0J7/0RRK0JZ/0RRK0KZ (ICD-10-CM) were examined. The exclusion criteria were as follows: (1) age < 18 years; (2) admission method: emergency, urgent, neonatal or another type of emergency room admission; and (3) pathological fractures of the humerus and clavicle.

### Data collection

Pulmonary complications were operationally defined in this study as.

(1) pneumonia (ICD-9-CM: 480/480.0/480.1/480.2/480.3/480.8/480.9/481/482/482.0/482.1/482.2/482.3/482.30/482.31/482.32/482.39/482.4/482.41/482.42/482.49/482.8/482.81/482.82/482.83/482.84/482.89/482.9/483/483.0/483.1/483.8/484/484.1/484.3/484.5/484.6/484.7/484.8/485/486;ICD-10-CM:J12/J12.0/J12.1/J12.2/J12.3/J12.8/J12.89/J12.9/J13/J14/J15/J15.1/J15.2/J15.20/J15.21/J15.211/J15.212/J15.29/J15.3/J15.4/J15.5/J15.6/J15.7/J15.8/J15.9/J16.0/J16.8/J17/J18/J18.0/J18.1/J18.2/J18.8/J18.9), (2) respiratory failure (ICD-9-CM: 51851, 51853, 51884, 51883, 51881, 77084; ICD-10-CM: J9582, J95821, j95822, J9600, j9602, J969, J961, J9601, J960, J962, J9610, J9612, J9620, P285, J96, J9611, J9691, J9690, J9692, J9621, J9622) and (3) PE (ICD-9-CM: 415.11, 415.19; ICD-10-CM: I260, I2699). (4) The term ‘any pulmonary complication’ was used to refer to the existence of one or more of the aforementioned issues affecting the pulmonary system. Patient characteristics, specifically age, gender and race, were studied. The analysis included outcome markers such as the mode of admission, type of insurance, length of stay (LOS), total cost of hospital stay, preoperative comorbidities and in-hospital mortality. ICD-9-CM and ICD-10-CM diagnostic codes were used to capture preoperative comorbidities that have the potential to be associated with pulmonary issues, as well as medical and surgical perioperative complications prior to the discharge of patients. According to NIS, a total of 29 variables indicate comorbidities, including AIDS, alcohol abuse, congestive heart failure, chronic pulmonary disease, coagulopathy, rheumatoid arthritis/collagen vascular diseases, chronic blood loss anaemia, depression, deficiency anaemia, uncomplicated diabetes, diabetes with chronic complications, drug abuse, fluid and electrolyte disorders, hypertension, hypothyroidism, liver disease, lymphoma, metastatic cancer, neurological disorders, obesity, paralysis, peripheral vascular disorders, psychoses, pulmonary circulation disorders, renal failure, solid tumour without metastasis, peptic ulcer disease, valvular disease and weight loss.

### Outcomes

Patient demographics, including age, gender and race, were evaluated. Outcome indicators, such as method of admission, LOS in the hospital, overall cost of hospitalization, comorbidities prior to surgery and in-hospital mortality, were analysed. The prevalence of pneumonia, respiratory failure and PE following TSA was the primary outcome. Secondary outcomes included variables such as LOS in the hospital, cost of admission, and inpatient mortality rate. Preoperative comorbidities that may be connected to pulmonary complications were separately collected using the ICD-9-CM and ICD-10-CM diagnosis codes.

### Data analysis

**Fig. 1:**
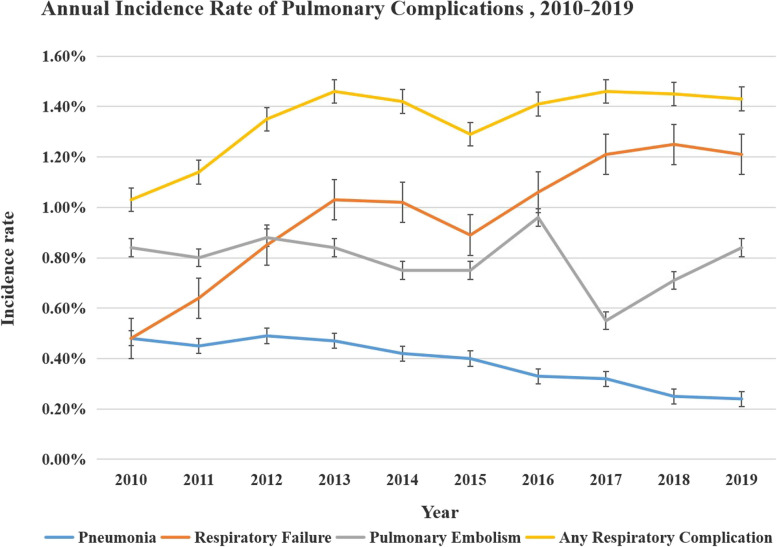
Annual incidence of pulmonary complications. The annual incidence of pneumonia, respiratory failure, and pulmonary embolism after elective total shoulder arthroplasty from 2010 to 2019 is shown above. The pooled incidence of any of the aforementioned complications is shown above by the graphlabeled “Any Pulmonary Complication.” The error bars show standard error of percent

The statistical plans were finalised before analysis was conducted. The Institutional Review Board no longer required a review of the analysis to be performed in this retrospective investigation due to the deidentification of patient files and their availability in the publicly accessible NIS surgical database. The hierarchical and cluster technique was utilized to calculate the estimates of the national incidence rate of these problems, and survey weights were applied in accordance with the NIS sample design. The yearly occurrence of pulmonary problems following TSA over the period spanning from 2010 to 2019 was assessed (Fig. 1).

Statistical analysis was conducted using SPSS (version 22.0). All tests were deemed statistically significant at a significance level of *p* < 0.05. The univariate relationships between independent factors and outcome variables were examined using chi-square test for categorical variables and Wilcoxon rank test for continuous variables. Binary logistic regression analysis was performed using stepwise regression to identify the independent risk factors for pneumonia, respiratory failure and PE. This analysis incorporated all factors such as demographic information supplied by NIS, characteristics of hospitals and preoperative comorbidities (Table 1). Previous studies in the area of NIS have often used large sample sizes, and the criterion for determining statistical significance at the alpha level was set at a threshold of P ≤ 0.05.

**Table 1:** Variables entered into the binary logistic regression analysis

Variables Categories	Specifc Variables
Patient demographics	Age (< 60 years and ≥ 60 years), sex (male and female), race (White, Black, Hispanic, Asian or Pacifc Islander, Native American and Other)
Hospital characteristics	Type of admission (non-elective, elective), bed size of hospital (small, medium, large), teaching status of hospital (nonteaching, teaching), location of hospital (rural, urban), location of the hospital (northeast, Midwest or north central, south, west)
Comorbidities	AIDS, alcohol abuse, defciency anemia, rheumatoid diseases, chronic blood loss anemia, congestive heart failure, chronic pulmonary disease, coagulopathy, depression, diabetes (uncomplicated), diabetes (with chronic complications), drug abuse, hypertension, hypothyroidism, liver disease, lymphoma, fuid and electrolyte disorders, metastatic cancer, neurological disorders, obesity, paralysis, peripheral vascular disorders, psychoses, pulmonary circulation disorders, renal failure, solid tumor without metastasis, peptic ulcer disease, valvular disease and weight loss

## Results

### Incidence of pulmonary complications after TSA

Between the years 2010 and 2019, a comprehensive analysis of the NIS database revealed the identification of 189,695 cases with TSA. A cohort of 2396 patients had pulmonary complications, resulting in an incidence rate of 1.4% (Table 2). A notable increase was found in the prevalence of pulmonary complications, with an increase observed from 4.8% in 2010 to 16.0% in 2019 (Fig. 1). The annual incidence of pulmonary complications following TSA demonstrated a considerable increase, as evidenced by a small yet statistically significant increases in the occurrence of respiratory failure (*P* < 0.001) and PE (*P* < 0.001). Meanwhile, the incidence of pneumonia throughout this time decreased (*P* < 0.001).

**Table 2:** Clinical characteristics and outcomes of patients with pulmonary complications after total Shoulder Arthroplasty

Comparison Group	Pneumonia	P	Respiratory Failure	P	Pulmonary Embolism	P	Any Pulmonary Complication	P	No Pulmonary Complication	P
**Total (*n* = count)**	626		1779		192		2396		171,377	
**Incidence (%)**	0.4		1		0.1		1.4		98.6	
**Age (average, yr)**	70 (63,76)	< 0.001	70(63,76)	< 0.001	70(63,76)	< 0.001	71(65,77)	< 0.001	70(63,76)	< 0.001
**Age group (%)**		< 0.001								
≤ 60	15.8		13.8		10.9		13.6		38.41	
61–70	29.7		33.6		33.9		33.0		34.71	
71–80	37.7		37		41.1		37.5		35.27	
≥ 81	16.8		15.6		14.1		15.9		11.24	
**Sex (% female)**	64.2	< 0.001	66.9	< 0.001	60.9	< 0.001	66.4	< 0.001	55.3	< 0.001
**Race (%)**		0.042		0.558		0.947		0.694		0.694
Caucasian	87.3		90.0		90.0		89.3		87.9	
African-American	6.3		4.6		5		5.0		4.5	
Hispanic	2.6		3.3		3.3		3.2		3.8	
Asian or Pacifi c Islander	1.0		0.4		0.6		0.5		5.0	
Native American	0.7		0.5		0.0		0.4		0.4	
Other^a^	2.1		1.2		1.1		1.6		1.6	
**Type of insure (%)**		< 0.001		< 0.001		0.173		< 0.001		< 0.001
Medicare	76.2		79.7		75.0		78.9		79.2	
Medicaid	3.7		3.6		3.6		3.7		3.8	
Private insurance	14.7		12.9		18.8		13.5		13.3	
Self-pay	0.8		0.4		1.0		0.5		0.7	
No charge	0.3		0.1		0.0		0.1		0.4	
Other^b^	4.3		3.2		1.6		3.3		2.6	
**CCI**	4(3,5)	< 0.001	5(4,7)	< 0.001	5(4,6)	< 0.001	5(4,7)	< 0.001	4(3,5)	< 0.001
**LOS (d)**	5(3,7)	< 0.001	3(2,5)	< 0.001	6(4,7)	< 0.001	4(2,6)	< 0.001	1(1,2)	< 0.001
**Cost (TOTCHG) **^**c**^	7.54 (5.38.,11.59)	< 0.001	7.98 (5.77,11.8)	< 0.001	8.2 (6.06,11.9)	< 0.001	7.77 (5.62,11.46)	< 0.001	5.72(4.2,7.93)	< 0.001
**Mortality (per 1000 cases)**	1.3	< 0.001	2.5	< 0.001	2.1	< 0.001	2.0	< 0.001	0	< 0.001

*Abbreviations*: *CCI* Charlson Comorbidity Index, *LOS* Length of Stay, *TSA* Total Shoulder Arthroplasty

^a^Other race includes Asian/Pacific Islander, Native American, and other categories

^b^Other primary payer includes self-pay, no insurance, no charge, missing, and other categories

^c^Unit of measurement is Ten thousand US dollars

### Patient demographics and hospital characteristics

Table 2 displays the patient demographic and hospital characteristics categorized on the basis of pulmonary complications. Of the 189,695 TSA procedures performed during 2010–2019, a pulmonary complication occurred following 1.4% (95% CI, 1.36%–1.46%) of TSA. Specifically, 0.4% (95% CI, 0.38%–0.42%) of TSA were followed by pneumonia, 1% (95% CI, 0.94%–1.22%) of TSA were followed by respiratory failure and 0.1% (95% CI, 0.08%–0.12%) of TSA were complicated by PE. The majority of patients affected by TSA were predominantly female, of Caucasian ethnicity and relied on Medicare as their primary source of payment. Those with pulmonary issues had a mean age ranging from 60 to 70 years (*P* < 0.001). A higher proportion of these individuals relied on Medicare as their primary payer than those without pulmonary complications (*P* < 0.001).

Table 2 presents the correlation between the development of pulmonary problems and several factors such as LOS, inpatient expenses, Charlson comorbidity index (CCI) and inpatient mortality. On average, patients who experience pulmonary issues after undergoing TSA tend to have an LOS that is about 3–5 times longer than those who do not experience such difficulties (*P* < 0.001).

**Fig. 2:**
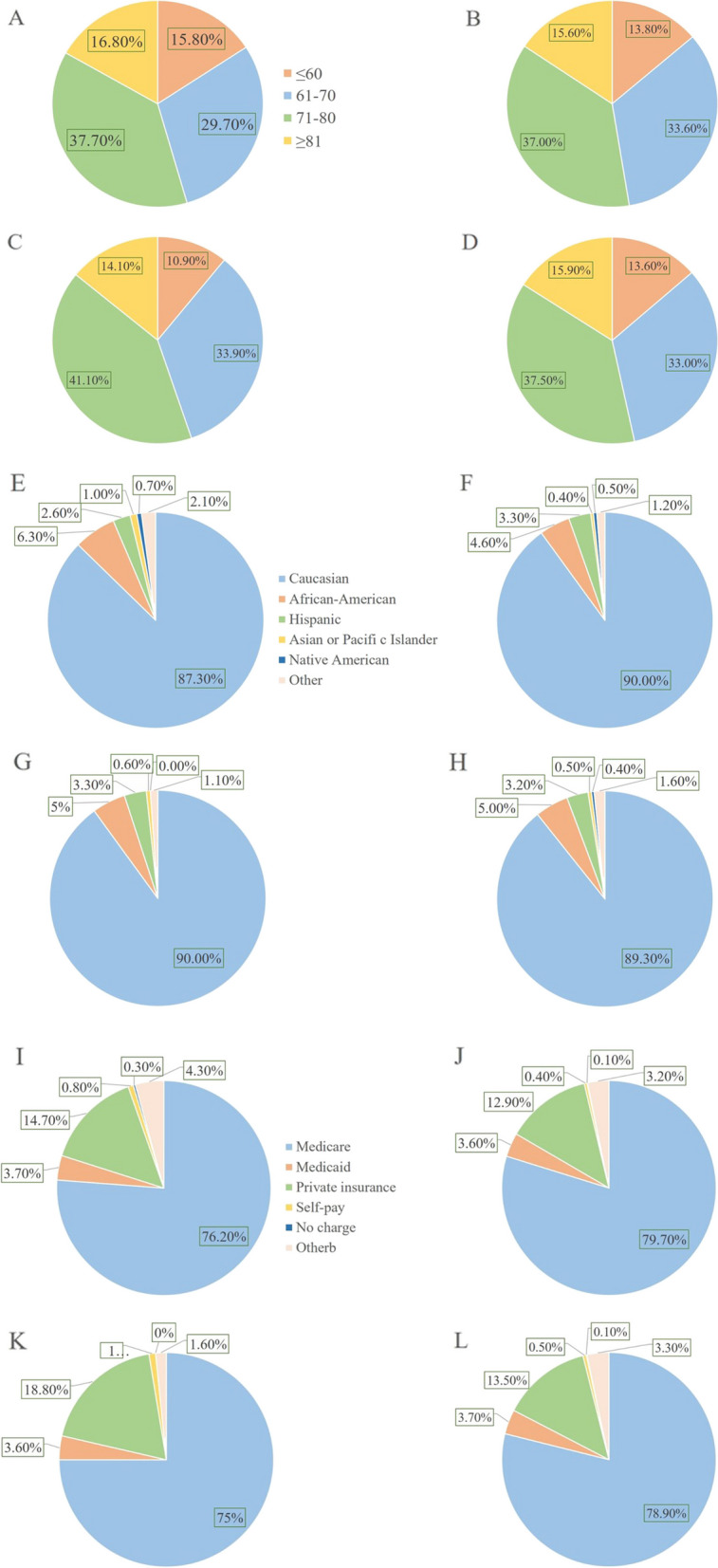
Patient demographics and hospital characteristics in the Patient of Pulmonary Complications After Total Shoulder Arthroplasty. **A** Age distribution analysis of Pneumonia. **B** Age distribution analysis of Respiratory Failure. **C** Age distribution analysis of Pulmonary Embolism. **D** Age distribution analysis of Any Pulmonary Complication**.E** Racial distribution analysis of Pneumonia. **F** Racial distribution analysis of Respiratory Failure. **G** Racial distribution analysis of Pulmonary Embolism. **H** Type of insure of Any Pulmonary Complication. **I** Type of insure of Pneumonia. **J** Type of insure of Respiratory Failure. **K** Type of insure of Pulmonary Embolism**.L** Type of insure of Any Pulmonary Complication

**Fig. 3:**
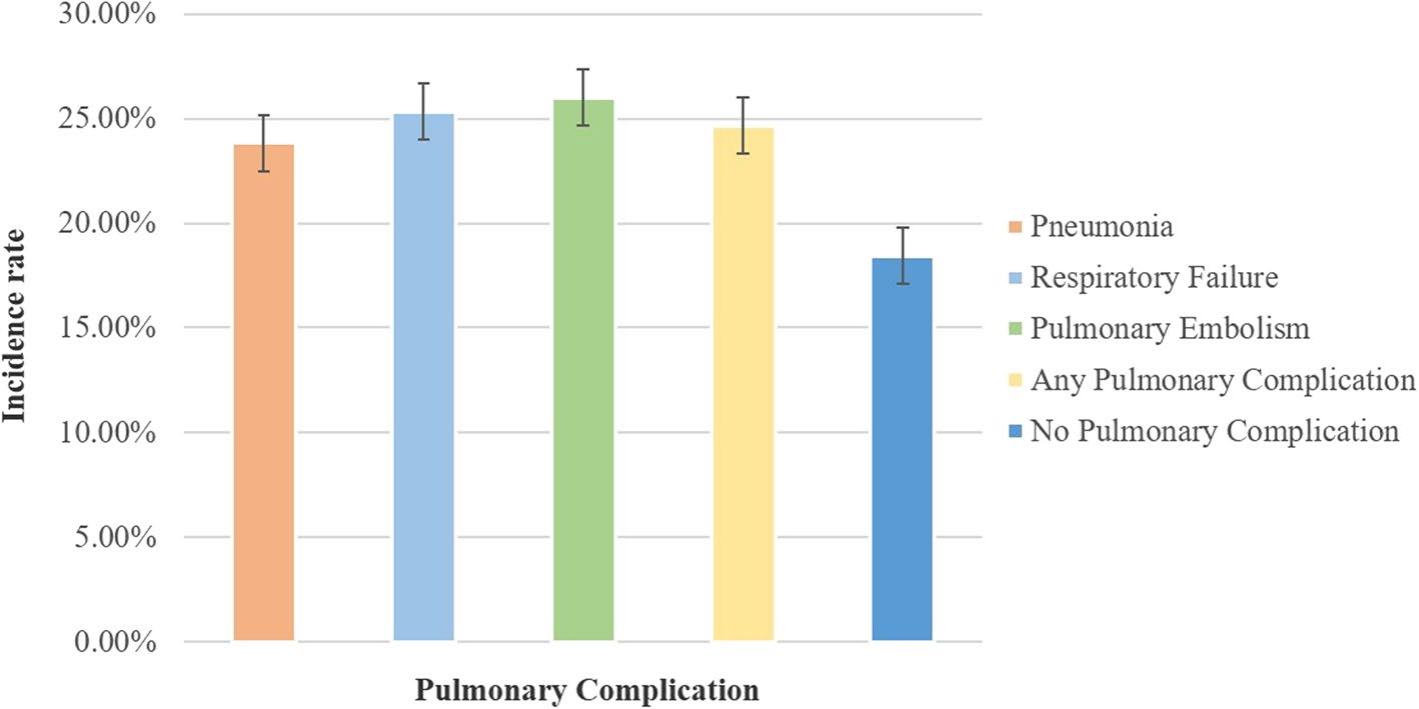
Patients costs of each of the five groups. Unit of cost is Ten thousand US dollars

PE conferred the greatest increase in inpatient LOS (TSA = 6; 99.9% CI, 4–7). Patient costs are shown in Figs. 2 and 3. Patients who encountered pulmonary difficulties incurred expenditures that were more than 30% higher those who did not have such issues (*P* < 0.001). PE exhibited the most significant increases in expenses for patients admitted to the hospital, with an increase of 8.8 Ten thousand US dollars (99.9% CI, 6.06–11.9, 10,000 US dollars). The odds of perioperative mortality following TSA was zero.

### Association between pulmonary complications and other preoperative comorbidities

Tables 3 and 4 show the multivariable regression results. The disorders most strongly related with pulmonary problems were shown to have the highest risk, including ulcer (adjusted odds ratio [AOR] = 9.43; 95% CI, 4.99–46.91), pulmonary circulation disorders (AOR = 9.01; 95% CI, 4.56–31.92), weight loss (AOR = 4.84; 95% CI, 2.15–10.88), fluid and electrolyte disorders (AOR = 3.55; 95% CI, 2.55–4.95), alcohol abuse (AOR = 1.56; 95% CI, 1.08–2.26), congestive heart failure (AOR = 3.09; 95%CI, 1.83—5.24), chronic pulmonary disease (AOR = 2.45; 95% CI, 1.60–3.75), deficiency anaemia (AOR = 1.56; 95% CI, 1.08–2.26), depression (AOR = 1.47; 95% CI, 1.03–2.11) and obesity (AOR = 1.46; 95% CI, 1.01–2.11). Pulmonary circulation disorders, weight loss, congestive heart failure and fluid and electrolyte disorders were the common diagnoses significantly associated with increased odds of pneumonia, respiratory failure, PE and any pulmonary complication. Valvular disease (AOR = 0.36, 95% CI, 0.18–0.72) and hypothyroidism (AOR = 0.91; 95% CI, 0.65–1.39) were shown to be strongly related to a lower likelihood of developing PE.

**Table 3:** Multivariable Analysis of Factors Associated With Pulmonary Complications After Total Joint after Shoulder Arthroplasty (Pneumonia and Respiratory Failure)

Variable	Pneumonia			Respiratory Failure		
	**OR**	**95%CI**	**P**	**OR**	**95%CI**	**P**
** ≥ 60**	0.66	(0.23,1.88)	0.44	1.83	(0.70,4.77)	0.21
**Sex (% female)**	0.75	(0.48,1.18)	0.21	0.83	(0.54,1.26)	0.38
**Race (%)**						
Caucasian	——	——	0.69	——	——	0.70
African-American	1.10	(0.39, 3.12)	0.85	0.43	(0.13,1.46)	0.18
Hispanic	1.49	(0.52, 4.23)	0.45	0.32	(0.04,2.37)	0.26
Asian or Pacifi c Islander	3.31	(0.44, 24.98)	0.24	0	0	0.99
Native American	3.11	(0.39, 24.68)	0.28	0	0	0.99
Other	0.790	(0.10, 5.86)	0.81	0.83	(0.17,4.01)	0.82
**Hospital bed size**						
Small	——	——	0.17	——	——	0.16
Medium	1.31	(0.68,2.50)	0.41	2.02	(0.97,4.21)	0.06
Big	0.82	(0.45,1.50)	0.53	1.73	(0.87,3.43)	0.11
**Hospital location**						
Teaching	0.98	(0.62,1.53)	0.93	1.10	(0.46,2.63)	0.81
Urban nonteaching	1.1	(0.41,2.94)	0.85	0.81	(0.53,1.24)	0.33
**Type of insure (%)**						
Medicare	——	——	< 0.05	——	——	< 0.05
Medicaid	3.34	(1.14,9.77)	< 0.05	2.01	(0.64,6.30)	0.22
Private insurance	1.30	(0.68,2.49)	0.42	0.80	(0.42,1.54)	0.52
Self-pay	3.87	(0.47,31.43)	0.20	7.72	(1.52,39.13)	< 0.05
No charge	18.38	(2.03,166.09)	< 0.05	20.43	(2.26,184.5)	< 0.05
Other	2.15	(0.73,6.34)	0.16	1.82	(0.68,4.90)	0.23
**CCI**	0.78	(0.19,3.25)	0.74	0.79	(0.52,1.20)	0.28
**Region of hospital**						
Northeast	——	——	0.61	——	——	0.46
Midwest	1.36	(0.63,2.93)	0.41	0.89	(0.44,1.79)	0.74
South	1.47	(0.74,2.93)	0.26	1.21	(0.67,2.21)	0.51
West	1.12	(0.50,2.49)	0.77	0.83	(0.41,1.69)	0.61
**AIDS**	0.00	(0.00, ——)	0.99	0.00	(0.00, ——)	0.99
**Alcohol abuse**	3.21	(1.16,8.89)	< 0.05	4.74	(1.91,11.7)	< 0.05
**Defciency anemia**	2.16	(1.32,3.54)	< 0.05	1.19	(0.71,1.9)	0.49
**Arthralgia**	1.55	(0.30,7.83)	0.59	1.82	(0.81,4.09)	0.14
**Chronic blood loss anemia**	2.91	(0.87,9.71)	0.08	1.83	(0.40,8.23)	0.42
**Congestive heart failure**	4.09	(0.87,19.15)	0.07	5.78	(2.97,11.26)	< 0.05
**Chronic pulmonary disease**	2.32	(0.51,10.42)	0.27	4.94	(2.81,8.68)	< 0.05
**Coagulopathy**	1.81	(0.73,4.46)	0.19	1.34	(0.52,3.43)	0.53
**Depression**	1.28	(0.74,2.21)	0.37	1.19	(0.72,1.9)	0.48
**Diabetes, uncomplicated**	1.21	(0.26,5.57)	0.81	0.95	(0.49,1.82)	0.88
**Diabetes with chronic complications**	1.54	(0.06,34.65)	0.78	3.37	(1.07,10.63)	< 0.05
**Drug abuse**	0.00	(0.00, ——)	0.99	0.00	(0.00, ——)	0.99
**Hypertension**	0.64	(0.40,1.02)	0.06	0.83	(0.53,1.31)	0.44
**Hypothyroidism**	1.12	(0.65,1.92)	0.67	1.01	(0.61,1.67)	0.96
**Liver**	1.60	(0.06,41.12)	0.77	0.00	(0.00, ——)	0.99
**Lymphoma**	0.00	(0.00, ——)	0.99	0.00	(0.00, ——)	0.99
**Fluid and electrolyte disorders**	4.02	(2.51,6.44)	< 0.05	3.60	(2.34,5.56)	< 0.05
**Metastatic cancer**	6.17	(0.09, 8.10)	0.53	30.23	(1.74,523.67)	< 0.05
**Neurological disorders**	2.79	(0.60, 13.00)	0.19	1.43	(0.63,3.25)	0.39
**Obesity**	1.04	(0.57,1.91)	0.87	1.64	(1.02,2.64)	< 0.05
**Paralysis**	0.00	(0.00, ——)	0.99	1.26	(0.12,12.73)	0.84
**Peripheral vascular disorders**	2.03	(0.39,2.56)	0.39	1.78	(0.73,4.34)	0.20
**Psychoses**	3.34	(0.17,6.87)	0.42	1.55	(0.39,6.09)	0.52
**Pulmonary circulation disorders**	2.69	(1.08,6.71)	< 0.05	7.40	(4.05,13.51)	< 0.05
**Renal failure**	2.16	(0.11,39.73)	0.60	3.42	(1.21, 9.66)	< 0.05
**Tumor**	0.00	(0.00, ——)	0.99	0.00	(0.00, ——)	0.99
**Ulcer**	0.00	(0.00, ——)	0.99	7.7	(3.72, 44.60)	< 0.05
**Valvular disease**	0.52	(0.18,1.51)	0.23	0.74	(0.34, 1.59)	0.44
**Weight loss**	6.26	(2.45,15.96)	< 0.05	6.00	(2.38, 15.13)	< 0.05

*Abbreviations*: *CCI* Charlson Comorbidity Index, *LOS* Length of Stay, *TSA* Total Shoulder Arthroplasty, *OR* Odds Ratio, *CI* Confidence Interval

**Table 4:** Multivariable Analysis of Factors Associated With Pulmonary Complications After Total Joint after Shoulder Arthroplasty ( Pulmonary Embolism and Any Respiratory Complication)

Variable	Pulmonary Embolism			Any Respiratory Complication		
	**OR**	**95%CI**	**P**	**OR**	**95%CI**	**P**
** ≥ 60**	1.03	(0.07,14.96)	0.98	0.97	(0.48,1.97)	0.95
**Sex (% female)**	2.69	(0.75,9.57)	0.12	0.90	(0.66,1.23)	0.53
**Race (%)**						
Caucasian	——	——	0.86	——	——	0.82
African-American	0.16	(0.01, 2.13)	0.16	0.58	(0.26,1.31)	0.19
Hispanic	0.00	(0.00, ——)	0.99	0.81	(0.32,2.05)	0.67
Asian or Pacifi c Islander	0.00	(0.00, ——)	0.99	1.59	(0.21,11.79)	0.64
Native American	0.00	(0.00, ——)	0.99	1.20	(0.15,9.56)	0.85
Other	0.00	(0.00, ——)	0.99	0.81	(0.23,2.85)	0.75
**Hospital bed size**						
Small	——	——	0.31	——	——	0.06
Medium	3.44	(0.58, 20.13)	0.17	1.69	(1.03,2.77)	0.03
Big	1.62	(0.31, 8.45)	0.56	1.21	(0.76,1.91)	0.40
**Hospital location**						
Teaching	2.75	(0.19, 38.37)	0.45	1.1	(0.56,2.14)	0.77
Urban nonteaching	0.66	(0.22, 1.94)	0.45	0.94	(0.69,1.28)	0.72
**Type of insure (%)**						
Medicare	——	——	0.99	——	——	< 0.05
Medicaid	0.43	(0.01,13.46)	0.63	2.32	(1.04, 5.16)	0.03
Private insurance	0.89	(0.18,4.34)	0.88	0.95	(0.60, 1.49)	0.82
Self-pay	0.00	(0.00, ——)	0.99	5.47	(1.41,21.13)	< 0.05
No charge	0.00	(0.00, ——)	0.99	8.08	(0.95,68.53)	0.06
Other	0.00	(0.00, ——)	0.99	1.65	(0.75,3.59)	0.20
**CCI**	0.70	(0.23, 2.06)	0.52	1.04	(0.76,1.43)	0.76
**Region of hospital**						
Northeast	——	——	< 0.05	——	——	0.32
Midwest	0.03	(0.003, 0.48)	< 0.05	0.86	(0.51,1.44)	0.57
South	1.19	(0.33, 4.23)	0.78	1.20	(0.78,1.85)	0.39
West	0.62	(0.13, 2.95)	0.55	0.93	(0.55,1.55)	0.78
**AIDS**	0.00	(0.00, ——)	0.99	0.00	(0.00, ——)	0.99
**Alcohol abuse**	0.00	(0.00, ——)	0.99	3.26	(1.53,6.94)	< 0.05
**Defciency anemia**	4.04	(1.15,14.17)	< 0.05	1.56	(1.08,2.26)	< 0.05
**Arthralgia**	1.06	(0.09,12.21)	0.95	1.07	(0.57,2.03)	0.81
**Chronic blood loss anemia**	0.00	(0.00, ——)	0.99	1.92	(0.65,5.64)	0.23
**Congestive heart failure**	2.71	(0.46,15.95)	0.26	3.09	(1.83,5.24)	< 0.05
**Chronic pulmonary disease**	1.40	(0.33,5.83)	0.64	2.45	(1.60,3.75)	< 0.05
**Coagulopathy**	6.0	(0.95,38.22)	0.06	1.87	(0.96,3.66)	0.06
**Depression**	3.79	(1.06,13.54)	< 0.05	1.47	(1.03,2.11)	< 0.05
**Diabetes, uncomplicated**	0.65	(0.15,2.82)	0.56	0.89	(0.55,1.43)	0.62
**Diabetes with chronic complications**	0.00	(0.00, ——)	0.99	1.50	(0.59,3.80)	0.38
**Drug abuse**	0.00	(0.00, ——)	0.99	0.00	(0.00, ——)	0.99
**Hypertension**	2.03	(0.60,6.84)	0.25	0.79	(0.57,1.10)	0.17
**Hypothyroidism**	0.2	(0.04,0.83)	< 0.05	0.91	(0.65,1.39)	< 0.05
**Liver**	0.00	(0.00, ——)	0.99	0.37	(0.07,1.96)	0.24
**Lymphoma**	0.00	(0.00, ——)	0.99	0.00	(0.00, ——)	0.99
**Fluid and electrolyte disorders**	0.66	(0.19,2.25)	0.51	3.55	(2.55,4.95)	< 0.05
**Metastatic cancer**	0.00	(0.00, ——)	0.99	2.91	(0.27,31.05)	0.37
**Neurological disorders**	0.16	(0.01,3.72)	0.25	1.34	(0.74,2.40)	0.32
**Obesity**	1.29	(0.36,4.65)	0.69	1.46	(1.01,2.11)	< 0.05
**Paralysis**	0.00	(0.00, ——)	0.99	0.33	(0.03,2.97)	0.32
**Peripheral vascular disorders**	0.00	(0.00, ——)	0.98	1.32	(0.64,2.72)	0.43
**Psychoses**	5.53	(0.22,37.99)	0.29	1.11	(0.41,2.95)	0.83
**Pulmonary circulation disorders**	9.83	(3.08,24.52)	< 0.05	9.01	(4.56,31.92)	< 0.05
**Renal failure**	4.031	(0.26,62.57)	0.31	1.61	(0.73,3.58)	0.23
**Tumor**	0.00	(0.00, ——)	0.99	0.00	(0.00, ——)	0.99
**Ulcer**	0.00	(0.00, ——)	1	9.43	(4.99,46.91)	< 0.05
**Valvular disease**	0.02	(0.00,0.22)	< 0.05	0.36	(0.18,0.72)	< 0.05
**Weight loss**	0.78	(0.04,17.07)	0.87	4.84	(2.15,10.88)	< 0.05

*Abbreviations: CCI* Charlson Comorbidity Index, *LOS* Length of Stay, *TSA* Total Shoulder Arthroplasty, *OR* Odds Ratio *CI* Confidence Interval

## Discussion

An epidemiological assessment of pulmonary problems following TSA and a comprehensive health economic analysis of such issues on a national scale within USA were conducted. On average, approximately one in every 72 cases of pneumonia, respiratory failure or PE was identified as complication during TSA operations. The occurrences of pneumonia, respiratory failure and PE following TSA were consistent with those previously reported (Bohl et al. 2017; Sloan et al. 2019). Ten et al. reported that 1.42% of THA procedures and 1.71% of TKA procedures were associated with the occurrence of pulmonary complications (Malcolm et al. 2020). However, reports about the prevalence of pneumonia, respiratory failure and PE following TSA are limited.

In contrast to the incidence of other significant perioperative complications, this study showed a decline alone in the yearly occurrence of pneumonia, a slight fluctuation in the annual incidence rate of PE, and a notable and persistent trend of increasing incidence of respiratory failure from 2010 to 2019. This trend has never been reported before in previous studies. For the trend of pneumonia, the possible explanations included improvements in the attached importance to perioperative risk of pulmonary complication, pulmonary hygiene, enhanced recovery after surgery, prevention for deep venous thrombosis, optimization of patients before surgery, procedures related to anaesthesia and surgery and care provided during the perioperative period (Wainwright et al. 2020; Becattini and Agnelli 2016; Bamgbade et al. 2007). Previously described displayed patients undergoing TSA as increasingly miscellaneous. This tendency may be reflected in the statistically significant but temporally minor increase in respiratory failure. Respiratory failure is widely recognized as a substantial patient safety signal. The monitoring and reporting of this complication have shown an upward trend due to its growing occurrence in comparison to other problems. However, it requires further investigation in the future (Scala and Pisani 2018).

The univariate analysis of the demographics indicated higher age (mainly aged 60–80 years) and female sex were associated with higher rates of respiratory complications after TSA. However, age and gender did not independently predict these complications when confounding demographic characteristics and comorbidities were considered. Research on lower extremities demonstrated that being female is an autonomous determinant for perioperative pulmonary complications subsequent to hip and knee arthroplasty (Young et al. 2015). According to Griffin et al., being a female is an autonomous risk factor for death resulting from perioperative PE in shoulder arthroplasty (Becattini and Agnelli 2016). The findings of the present study align with those of previous research, indicating a higher prevalence of shoulder arthroplasty amongst women than males. However, no relationship was found between gender and pulmonary complications after TSA. Several studies have reported that hormone replacement treatment in postmenopausal women is associated with an increased risk of PE (Kaptein et al. 2021). Surgeons must exercise due diligence in attending to this particular aspect whilst conducting surgeries on these specific patients.

The adjusted analysis indicated pulmonary circulation disorders, weight loss, fluid and electrolyte disorders, alcohol abuse, congestive heart failure, chronic pulmonary disease, deficiency anaemia, depression and obesity were associated with pulmonary complications after TSA. Pulmonary circulation problems and congestive heart failure are potential causes of pulmonary complications resulting from pulmonary venous stasis and physical dependency (Kaptein et al. 2021). Why weight loss, obesity and fluid imbalances are strongly linked with postoperative pulmonary problems in the current research and earlier publications is not clear. According to Bohl et al., individuals with a body mass index (BMI) below 24 kg/m^2^ have a 50% higher likelihood of developing pneumonia following TJA in comparison to those with a BMI over 35 kg/m^2^ (Cogan et al. 2023; Griffin et al. 2014; Gupta et al. 2015). The research of Johnson et al. on 151,700 National Surgical Quality Improvement Program (NSQIP) patients undergoing vascular and general surgery showed similar results of patients with weight loss > 10% having a considerably higher risk of postoperative respiratory failure (Lung et al. 2019). Weight loss and fluid imbalances may potentially serve as manifestations of chronic illness.

This study suggested obesity and risk of respiratory failure had a positive association, but the degree was less than that in weight loss. In their investigation on medical problems following TSA amongst patients with and without obesity, Gupta et al. discovered a heightened incidence of respiratory failure, cardiac events, renal failure and blood transfusion amongst individuals with BMI over 35 kg/m^2^ (Bamgbade et al. 2007). Charles J et al. revealed that the incidence of medical complications exhibited a gradual increase with higher BMI. Specifically, the rates of PE complications were found to be 159% higher with each increment in BMI (Jiang et al. 2016). The proposed aetiologies of respiratory failure could be derived from obesity, because obesity hypoventilation increases the risk of anaesthesia, difficult exposure increased operative time, increased blood loss necessitating transfusions and reduced cardiovascular reserve to cope with intraoperative physiological stress. The number of individuals with obesity that requires TSA is increasing. Surgeons and anaesthesiologists are advised to provide proper guidance to patients with obesity on the potential perioperative risks associated with medical complications.

Dimick et al. observed that respiratory problems, such as pneumonia and respiratory failure, accounted for the highest attributable cost (52,466 US dollars) across various complications, including respiratory, cardiovascular, thromboembolic, and infectious complications, following noncardiac surgery at a solitary private-sector medical facility (Masuku et al. 2022). In the present study, the development of a pulmonary complication following TSA resulted in a significant increase in hospitalization expenditures, ranging from 50 to 70% on average. Additionally, the LOS in the hospital was found to be 3–6 times longer than in cases without such complications. The statistical results of PE were the highest, and the most relevant risk factors for PE were pulmonary circulation disorders, deficiency anaemia and depression. Research showed that reduction in the incidence of PE can be intervened by thrombophlaxis and potential anticoagulant therapy (Willis et al. 2009). Accordingly, understanding the relevant risk factors before surgery, risk stratification and proper management and selecting the adequate level of intensity of care for each admitted patient are critical to improve outcomes. Multiple research on PE indicated that the implementation of pre-screening, risk stratification and appropriate management strategies is of utmost importance in enhancing patient outcomes (Kaptein et al. 2021). No significant correlation was observed between pulmonary complications and mortality in the present study, may be because follow-up data were not incorporated. Further investigation may be necessary.

The NIS database, being the biggest publicly available inpatient database that encompasses all payers, is considered to be a reliable reflection of national trends with low influence from selection bias (Magill et al. 2014). Consequently, the prevailing patterns of pulmonary problems following TSA across USA were depicted. However, this study has some limitations. The main limitation is inherent to NIS database studies, such as lack of clinical number of cases, coding inaccuracies and lack of follow-up data. The dataset consisting of medical billing data only may be prone to coding mistakes, thereby affecting the reliability of the findings. Additionally, the use of multivariable models may not be sufficient to effectively explain the observed patterns in the development of pulmonary complications. After 2014, systematic billing codes changed from ICD-9 to ICD-10. So, the accurate entering of ICD-9 and ICD-10 codes appears particularly important.

The NIS database is specific to USA and may not accurately reflect the healthcare practices, patient demographics or disease prevalence in other countries. For example, healthcare practices, patient behaviour and treatment protocols vary significantly between USA and China. The NIS data may not capture the unique cultural and social factors influencing patient management in China, thereby limiting the generalizability of the findings to the Chinese context. These limitations suggest that whilst insights from NIS may be informative, they cannot be directly applied to patient management in China without significant adaptation and consideration of local contexts. Although the NIS database is not directly applicable to the management of Chinese patients, its concepts and structure can be used to build a patient management database suitable for the Chinese medical system.

The need to assess several potential interactions in these studies increases the likelihood of obtaining misleading findings. Although reducing the *p*-value partially mitigates this risk, it cannot be completely removed. Consequently, these studies cannot be utilized as definitive evidence of specific connections. However, they can be employed for exploratory objectives in identifying potential interactions. Insufficient follow-up data could result in an underestimation of postoperative pulmonary issues on an individual basis. The occurrence of pulmonary complications following TSA is infrequent. Due to the significant increase in costs, LOS in the hospital and mortality risk, managing pulmonary complications in a comprehensive manner, with special focus on PE, is essential.

## Data Availability

This study is based on data provided by Nationwide Inpatient Sample (NIS) database, part of the Healthcare Cost and Utilization Project, Agency for Healthcare Research and Quality. The NIS database is a large publicly available all-payer inpatient care database in the United States. Therefore, individual or grouped data cannot be shared by the authors.

## Abbreviations

TSA: Total Shoulder Arthroplasty
NIS: National Inpatient Sample
CCI: Charlson Comorbidity Index
LOS: Length of Stay
OR: Odds Ratio
CI: Confidence Interval
